# Supplementary material for: The causal role of circulating inflammatory markers in osteoporosis: a bidirectional Mendelian randomized study
Source: Front Immunol. 2024 Jul 18;15:1412298. doi: 10.3389/fimmu.2024.1412298 (PMC11291241; doi:10.3389/fimmu.2024.1412298)
Supplement: Supplementary file 1 [file DataSheet_1.zip › supplementary material/OSTEOPOROSIS-CIM/GCST90274796.tsv.gz_leave_one_out.pdf]

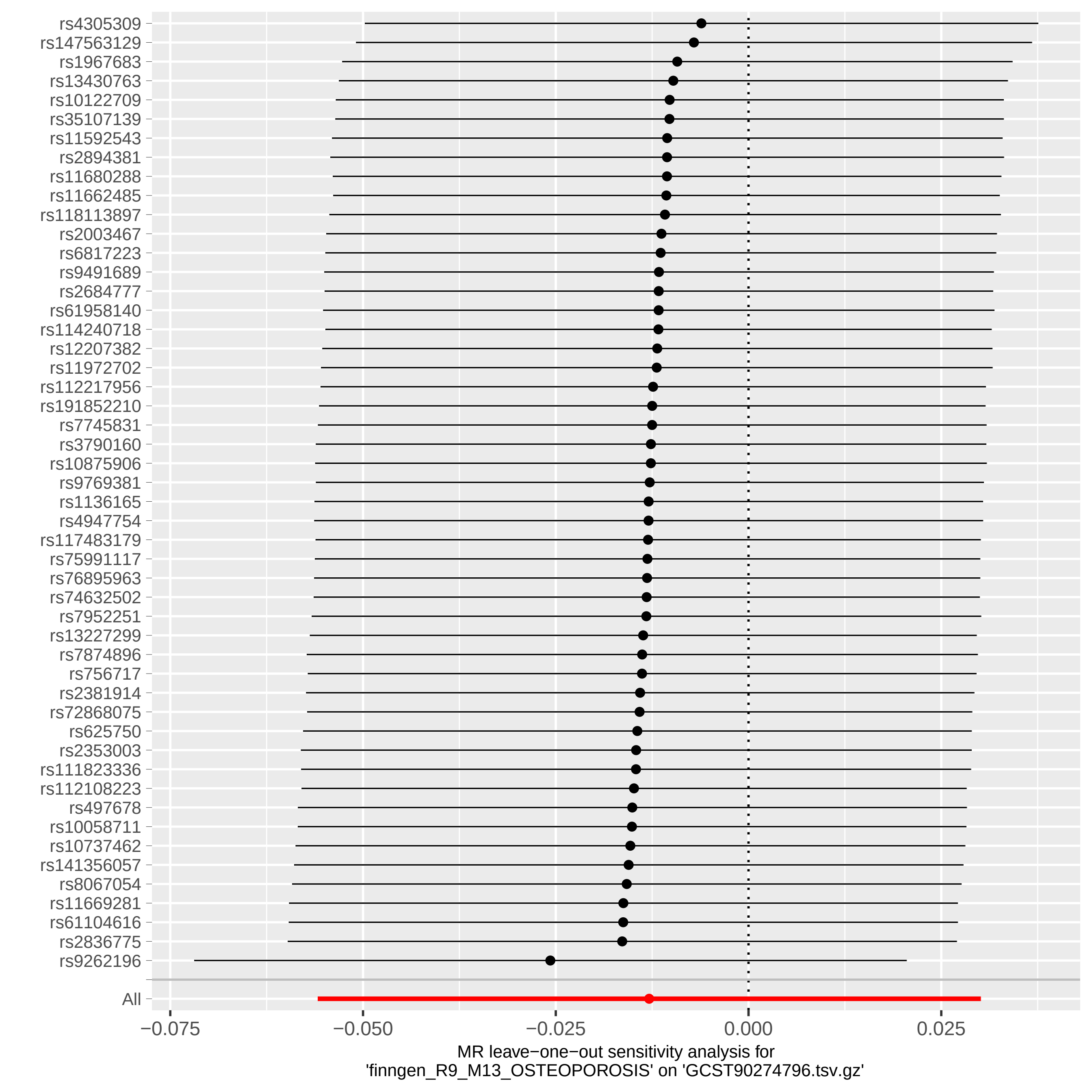
rs4305309
rs147563129
rs1967683
rs13430763
rs10122709
rs35107139
rs11592543
rs2894381
rs11680288
rs11662485
rs118113897
rs2003467
rs6817223
rs9491689
rs2684777
rs61958140
rs114240718
rs12207382
rs11972702
rs112217956
rs191852210
rs7745831
rs3790160
rs10875906
rs9769381
rs1136165
rs4947754
rs117483179
rs75991117
rs76895963
rs74632502
rs7952251
rs13227299
rs7874896
rs756717
rs2381914
rs72868075
rs625750
rs2353003
rs111823336
rs112108223
rs497678
rs10058711
rs10737462
rs141356057
rs8067054
rs11669281
rs61104616
rs2836775
rs9262196
All
−0.075
−0.050
−0.025
0.000
0.025
MR leave−one−out sensitivity analysis for
'finngen_R9_M13_OSTEOPOROSIS' on 'GCST90274796.tsv.gz'
